# Effect of Fiber Surface Characteristics on the Interfacial Properties of T1100-Grade Carbon Fiber Bismaleimide Composites

**DOI:** 10.3390/polym18070887

**Published:** 2026-04-05

**Authors:** Tianshu Li, Fenghui Shi, Weihan Wang, Hongchen Yan, Xiangyu Xu, Baoyan Zhang

**Affiliations:** AVIC Manufacturing Technology Institute Composite Technology Center, Beijing 101300, China; buaalitianshu@126.com (T.L.);

**Keywords:** T1100-grade carbon fiber, surface characteristics, sizing agent, interfacial region, interfacial shear strength

## Abstract

To clarify the effect of surface characteristics on the interfacial properties of T1100-grade carbon fiber (CF)/bismaleimide (BMI) composites, three CFs (F1, F2, and F3) with different surface treatments and sizing agents were studied. Surface physicochemical properties and sizing–resin reaction behavior were characterized; nano-infrared spectroscopy was innovatively used to quantify interfacial structure. The correlation among surface features, interfacial structure, and mechanical properties was established. All dry-jet wet-spun T1100 CFs show smooth surfaces with similar roughness, and mechanical interlocking contributes little to interfacial adhesion. F3 possesses the highest active carbon, oxygen content, and epoxy value. Its sizing agent exhibits strong reactivity with BMI, forming a ~200 nm thick interface and the highest interfacial shear strength (IFSS) of 95.9 MPa. Constructing a “thick and strong” interface promotes shear failure from brittle to tough, significantly enhancing 90° tensile and interlaminar shear strength (ILSS). This work provides guidance for interface design and engineering applications of T1100/BMI composites in aerospace primary load-bearing structures.

## 1. Introduction

Since Toray Industries, Inc., officially launched T1100 carbon fiber in 2014, it has rapidly become a potential reinforcement material for composite materials used in the main load-bearing structures of aerospace vehicles due to its higher strength and modulus [1,2,3,4]. However, unlike T800-grade CFs, which can be produced by both wet-spinning and dry-jet wet-spinning processes, the industrial large-scale production of T1100-grade CFs (to meet the main technical indicators: strength > 6600 MPa and modulus > 324 GPa) currently relies solely on dry-jet wet spinning. This results in a smooth fiber surface with few grooves; meanwhile, higher carbonization temperatures are required during fiber production to achieve a higher modulus, leading to a more compact graphite crystal structure and stronger surface inertness [2]. These two characteristics render the interfacial matching between T1100-grade CFs and resins more challenging, imposing higher requirements for the development of composite materials [5,6,7].

The interfacial theories of carbon fiber-reinforced polymer (CFRP) mainly include mechanical interlocking [6,8], chemical bonding [9,10], physical wetting [11,12], diffusion theory [13], and transition layer theory [14]. Due to the smooth surface of dry-jet wet-spun CFs, mechanical interlocking plays a minor role in the interfacial matching of T1100-grade CFs. Surface oxidation treatment and coating modification are the main engineering methods to improve the surface characteristics of smooth CFs [15]. Regarding research on surface oxidation treatment [16,17], Hui et al. [18] reported that grafting PA6 onto oxidized carbon fibers effectively improves interfacial adhesion and significantly enhances the mechanical properties of CF/PA12 composites. Zhang et al. [19] reported that a double alternant “rigid–flexible” gradient structure constructed on carbon fibers via grafting polydopamine and polyethyleneimine can significantly enhance interfacial and mechanical properties of CF/epoxy composites, providing a useful strategy for developing high-strength and high-toughness composites. Sun et al. [20] reported that in situ-polymerized PA6 coating on carbon fibers solves the incompatibility of commercial sizing agents and significantly improves the interfacial properties of CF/polyamide composites. A Kaveh et al. reported that electrochemical oxidation effectively introduces carboxylic groups, increases surface oxygen content and porosity, and significantly improves the surface chemistry and morphology of PAN-based carbon fibers. A Kaveh et al. [21] demonstrated that electrochemical oxidation can increase carboxyl groups, oxygen content, and surface porosity, thus significantly improving the surface chemistry and morphology of PAN-based carbon fibers. He et al. [22] oxidized the CF surface in 0.5 mol/L phosphoric acid solution at a current density of 0.2 A using CF as the working electrode and platinum as the auxiliary electrode, generating more active sites and significantly increasing the concentration of active groups and roughness on the CF surface. For T1100-grade CFs, further control of current density and electrolyte concentration is required to achieve optimal surface oxidation treatment.

Coatings for improving interfaces mainly include resin-based sizing agents, coupling agent coatings, and nanomaterial coatings. Resin-based sizing agents can enhance interfacial adhesion between CFs and the matrix and reduce interfacial defects; common sizing agents include epoxy resin, polyester, polyamide, and polyimide [23,24,25,26]. Ren et al. [27] sized CFs with E51 and E20 epoxy resins and found that epoxy sizing agents can act as catalysts to eliminate residual thermal stress and defects at the interface, improving ILSS. Coupling agent coatings can enhance interfacial compatibility; Hu et al. [28] covalently grafted MXene nanosheets onto the CF surface via silane coupling agents, significantly increasing the surface free energy, roughness, and polar group content of CFs and improving interfacial compatibility. Zhu et al. [29] modified CFs by immersing them in a solution of the silane coupling agent 3-aminopropyltriethoxysilane, improving the interfacial bonding between CFs and the matrix. Nanomaterial coatings involve depositing nanoparticles (e.g., nano-silica [30], graphene oxide [31], carbon nanotubes [32], and MXene nanosheets [33]) onto the CF surface to form modified coatings, using the same application method as resin-based sizing agents. Nanoparticles can increase the contact area and active sites between fibers and the matrix, thereby enhancing the interfacial and mechanical properties of CFRP. Currently, there are no targeted sizing agents or coating materials for modifying the surface characteristics of T1100-grade CFs, and suitable sizing agents need to be tailored to their surface properties.

To conduct in-depth research on composite interfaces, scholars have developed various precise characterization techniques to reveal interfacial micro-characteristics [34], including scanning electron microscopy (SEM) [35], transmission electron microscopy (TEM) [36], atomic force microscopy (AFM) [37], and Raman spectroscopy [38]. For example, Zhang et al. [35] observed the fiber/matrix debonding behavior by examining the fracture surfaces of carbon nanotube-grafted CF polymer composites and summarized three toughening mechanisms: crack bridging, microcrack formation, and carbon nanotube pull-out.

Compared with mature grades such as T800, research on the interface of T1100-grade CFs is still in its initial stage. To date, no study has focused on the interfacial structure of T1100-grade CFs with a smooth surface and high graphitic crystallinity prepared by the dry-jet wet-spinning process, nor has any research revealed the dominant factors influencing the interfacial strength of T1100-grade CF composites. Meanwhile, the multi-scale relationship among the surface characteristics of T1100-grade CFs, the fiber/resin interfacial structure, and the composite properties remains unclear. To address the above issues, this study innovatively employs nano-infrared spectroscopy to analyze the thickness and functional group concentration of the interfacial region in composites reinforced with carbon fibers containing different sizing agents. The reaction characteristics and functional group evolution between the sizing agent and BMI resin are monitored via in situ infrared spectroscopy. Furthermore, the interfacial microstructure of T1100-grade CFs and the main factors affecting it are clarified, and the law governing how interfacial strength is influenced by interfacial region thickness and functional group concentration is determined. Finally, the relationship among the surface characteristics, interfacial microstructure, and macroscopic mechanical properties of T1100-grade CFs is established by characterizing the 90° tensile strength, interlaminar shear strength, and fracture morphology of the composites. The results provide theoretical and experimental references for the regulation of the surface microstructure of T1100-grade CFs, the design of sizing agents, and their interfacial matching with bismaleimide resin.

## 2. Experimental Materials and Methods

### 2.1. Materials

Three types of T1100-grade CFs with different surface treatment states and sizing agents were used, labeled as F1, F2, and F3. All three fibers adopt water-based sizing agents formulated on the basis of epoxy resin. The sizing agent for F1 uses cycloaliphatic epoxy resin and contains polyether surfactant and no curing agent, so it cannot undergo a self-curing reaction. The sizing agent for F2 uses bisphenol A epoxy resin and is incorporated with a large amount of polyether surfactant and a small amount of amine curing agent, which enables slight self-curing but has a low overall epoxy value. The sizing agent for F3 uses bisphenol A epoxy resin and contains less polyether surfactant than F2 but is added with a higher content of amine curing agent, allowing for sufficient self-curing reaction and a higher overall epoxy value. All fibers were provided by Weihai Tuozhan Fiber Co., Ltd. (Weihai, China) The BMI resin used in this study is a self-made high-toughness resin system, mainly composed of diallyl bisphenol A (DABPA), 4,4′-bismaleimidodiphenylmethane (BDM), and related toughening agents, labeled as BMI.

### 2.2. Preparation of Composites

T1100-grade carbon fiber/BMI resin prepregs were prepared by the hot-melt method at a compounding temperature of 85~95 °C and a coating speed of 2~4 m/min. The prepared prepregs had an areal density of 200 ± 5 g/m^2^ and a resin content of 33 ± 2%. The lay-up of nano-infrared composite samples was 0_[16]_, the lay-up of 90° tensile composite samples was 90_[16]_, and the lay-up of ILSS samples was 0_[24]_. All composite samples were cured via the autoclave process with a curing cycle of 180 °C for 2 h + 200 °C for 6 h and a curing pressure of 0.6 MPa (as shown in Figure S1).

### 2.3. Performance Characterization Methods

The surface morphology of T1100-grade CFs and the fracture surfaces of their composites were observed using an FEI Quanta 450 FEG (Thermo Fisher Scientific, Hillsboro, OR, USA) field-emission scanning electron microscope at an accelerating voltage of 20 kV and a working distance of 9~11 mm. The surface roughness of CFs was characterized using a Dimension ICON atomic force microscope (AFM, Bruker Corporation, Billerica, MA, USA), and the roughness values were calculated via Nano Scope Analysis software (version 3.0) with an analysis area of 3 μm × 3 μm. The Raman spectra of the surface and cross-section of CFs were tested using a JY LabRAM Raman spectrometer (HORIBA Jobin Yvon, Longjumeau, France) with a 632 nm laser source, a scanning range of 800~2000 cm^−1^, and a scanning time of 2 min. The surface element content of CFs was determined using a Thermo Scientific ESCALAB 250XI X-ray photoelectron spectrometer (XPS, Thermo Fisher Scientific, Waltham, MA, USA) with a monochromatic Al Kα X-ray source. Wide-scan spectra were first collected, followed by high-resolution narrow-scan spectra of the C 1s peak. Using the peak at 284.6 eV as the reference, curve fitting of the C 1s spectrum was performed to investigate the binding energy of carbon elements. The surface epoxy value of CFs was tested by chemical titration: 1~2 g of CF was cut into pieces and ultrasonically oscillated in carbon tetrachloride (CCl_4_) for 10 min, and after adding an indicator, a 0.1 mol/L perchloric acid standard titrant was used for titration to calculate the epoxy value. The surface energy of CFs was analyzed using an IGC-SEA inverse gas chromatography surface energy analyzer (IGC, Surface Measurement Systems Ltd., Greenford, UK), and the polar and dispersive components were calculated separately.

The chemical reaction characteristics of the sizing agents, BMI resin, and 1:1 mixtures of sizing agents and BMI resin were tested using a Discovery DSC250 differential scanning calorimeter (DSC, TA Instruments, New Castle, DE, USA) at a heating rate of 10 °C/min and a test temperature range of 50 °C to 400 °C. The in situ Fourier transform infrared (FTIR) spectra of the sizing agents and their 1:1 mixtures with BMI resin were recorded using a Nicolet IS50 FTIR spectrometer (Thermo Fisher Scientific, Waltham, MA, USA) at a heating rate of 20 °C/min from room temperature to 200 °C, monitoring the changes in infrared spectra during heating.

The infrared characteristics of the composite interfacial region were analyzed using a Bruker Nano IR3 nano-infrared analysis system (Bruker Corporation, Santa Barbara, CA, USA) with a line scan interval of 40 nm and an infrared spectrum scanning range of 800~3600 cm^−1^. The interfacial shear strength (IFSS) of the CF/BMI resin composites was characterized using an HM410 composite interface tester (TOEI SANGYO Co., Ltd., Tokyo, Japan) at a loading rate of 0.05 mm/s, with an embedded length of 50~100 μm, and the IFSS was calculated as follows:

$$\tau_{IFSS}=\frac{F}{\pi dl}$$

where $\tau_{IFSS}$ is the interfacial shear strength (MPa), *F* is the maximum debonding force (N), *d* is the diameter of the CF monofilament (μm), and *l* is the embedding length of the CF in the resin bead (mm). Ten valid data points were tested, and the average value was calculated. The 90° tensile strength of different CF/BMI composites was tested in accordance with ASTM D3039 [39], with specimen dimensions of 175 mm × 25 mm × 2 mm and a crosshead speed of 1 mm/min. Interlaminar shear strength (ILSS) was tested in accordance with ASTM D2344 [40], with specimen dimensions of 20 mm × 6 mm × 3 mm, a span length of 12 mm, and a crosshead speed of 1 mm/min.

## 3. Results and Discussion

### 3.1. Surface Morphology and Physical Properties of Different T1100-Grade CFs

**Figure 1:**
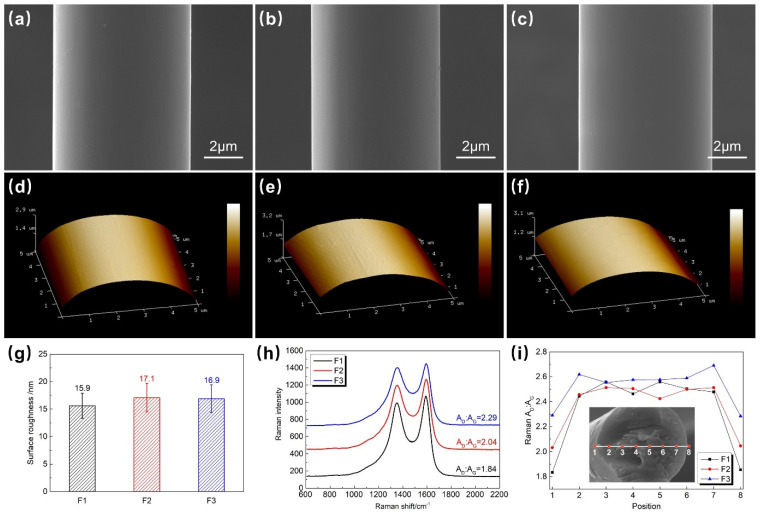
Surface SEM images of (**a**) F1, (**b**) F2, and (**c**) F3; AFM images of (**d**) F1, (**e**) F2, and (**f**) F3; (**g**) surface roughness of the three CFs; (**h**) surface Raman spectra of the three CFs; (**i**) cross-sectional Raman spectroscopy line scanning of the three CFs.

The surface morphology and graphite crystallization characteristics of three types of T1100-grade CFs were analyzed. As shown in Figure 1a–c, all three CFs exhibit a smooth surface morphology typical of dry-jet wet spinning, with no obvious grooves. The surfaces of F1 and F2 have a small amount of uneven sizing agent. Figure 1d–f show the AFM images of the three fibers, and only a few fine grooves generated by electrochemical treatment are observed on the fiber surfaces. As shown in Figure 1g, the surface roughness of the three fibers is similar, all approximately 16~17 nm, indicating similar surface physical groove characteristics. The smooth surface morphology and small, similar surface roughness of the three CFs lead to similar and low contributions of mechanical interlocking to the interfacial bonding between CFs and the resin. Physical wetting and chemical bonding may be the main factors affecting the interfacial properties of different T1100-grade CFs.

Raman spectroscopy analysis was performed on the surface and cross-section of the three CFs. The ratio of the area of the D peak (around 1350 cm^−1^) to the area of the G peak (around 1580 cm^−1^) (A_D_:A_G_) was used to characterize the graphitization degree of the CF surface, with a smaller ratio indicating a higher graphitization degree. The variation is attributed to the difference in carbonization temperature, where F1 fiber exhibits the highest carbonization temperature, followed by F2 fiber, and F3 fiber has the lowest. A higher carbonization temperature contributes to the improved graphitization degree of the carbon fiber surface. As shown in Figure 1h, the A_D_:A_G_ ratio increases gradually from F1 to F3, indicating a gradual decrease in the surface graphitization degree and a gradual reduction in surface inertness. In Figure 1i, cross-sectional Raman spectroscopy line scanning shows that the internal A_D_:A_G_ ratio of F1 and F2 is higher than that of F3 fibers, which may be related to the higher carbonization temperature during their production. In summary, due to the smooth surface and similar surface roughness of the fibers, this study basically ignores the contribution of mechanical interlocking to interfacial properties and focuses on analyzing the effects of sizing agents and fiber surface chemical characteristics on interfacial properties.

### 3.2. Surface Chemical Characteristics of Different T1100-Grade CFs

**Figure 2:**
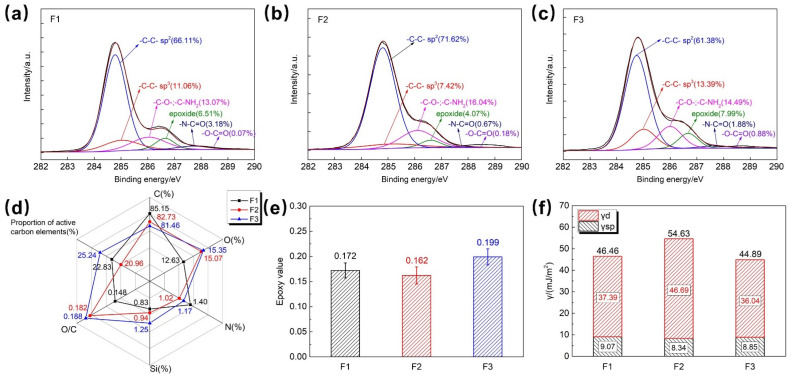
C_1s_ curve fitting of (**a**) F1, (**b**) F2, and (**c**) F3; (**d**) XPS surface element content of the three CFs; (**e**) surface epoxy value of the three CFs; (**f**) surface energy comparison of the three CFs.

The surface chemical composition and chemical characteristics of different CFs were analyzed. Figure 2a–c show the curve-fitted C_1s_ XPS spectra of the three CFs. The content of active carbon atoms in F1, F2, and F3 is 22.83%, 20.96%, and 25.24%, respectively. Among them, the content of active carbon atoms related to amino and hydroxyl groups is 13.07%, 16.04%, and 14.49%, respectively, and the content of active carbon atoms related to epoxy groups is 6.51%, 4.07%, and 7.99%, respectively. F3 has the highest content of active carbon atoms and epoxy groups. F2 has the lowest content of active carbon atoms and epoxy groups, which may affect interfacial chemical reactions. However, F2 has the highest content of active carbon atoms related to amino and hydroxyl groups, which is beneficial to improving surface polarity and enhancing the wetting between fibers and resin. Figure 2d shows the element content and O/C ratio of the three fibers. F1 has the highest surface C content and the lowest O content, followed by F2. F3 has the highest O content and the lowest C content, with a surface O/C ratio of 0.188, which is conducive to the wetting between fibers and resin.

The surface epoxy value of the fibers was analyzed. As shown in Figure 2e, the trend of the epoxy value of the three CFs (calculated based on 1% sizing amount) is consistent with the content of active carbon atoms of epoxy groups in the C_1s_ curve-fitting results. Figure 2f shows the surface energy of the three CFs tested by IGC. Although F2 has the lowest content of active carbon atoms, it exhibits the highest surface energy. This phenomenon is probably caused by the relatively high content of polyether surfactants in its sizing agent, as surfactants can enhance the surface energy of CFs. However, the polar component of surface energy, which contributes more significantly to the wetting between resin and fiber, is only 8.34 mJ/m^2^, the smallest among the three fibers. F3 has the lowest surface energy, and its polar component of surface energy is slightly lower than that of F1 (9.07 mJ/m^2^), at 8.85 mJ/m^2^. In summary, F3 has the highest content of active carbon atoms, surface oxygen content, O/C ratio, and surface epoxy value, which is conducive to the wetting between fibers and resin and the formation of good interfacial bonding.

### 3.3. Reaction Characteristics of Sizing Agents of Different T1100-Grade CFs with Resin

**Figure 3:**
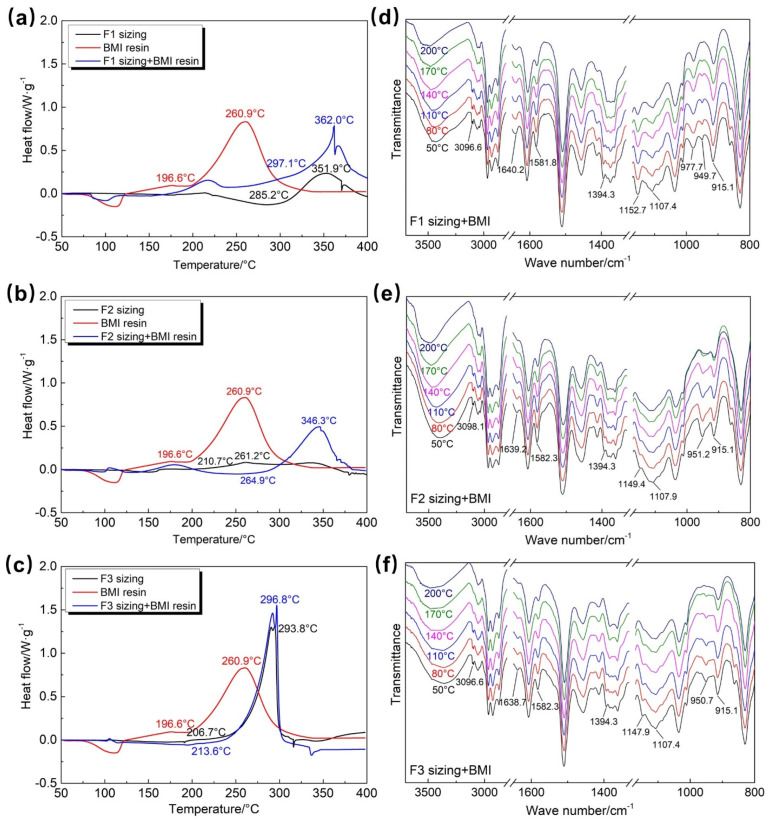
DSC curves of (**a**) F1, (**b**) F2, and (**c**) F3 fiber sizing agents, BMI resin, and their 1:1 mixtures with BMI resin; in situ FTIR spectra of (**d**) F1, (**e**) F2, and (**f**) F3 fiber sizing agents and their 1:1 mixtures with BMI resin.

The reaction characteristics of different sizing agents, BMI resin, and their 1:1 mixtures were analyzed by DSC, and the results are shown in Figure 3a–c and Table 1. The BMI resin starts to react at approximately 197 °C and reaches the reaction peak temperature at approximately 261 °C. The F1 sizing agent does not undergo a self-curing reaction before 285 °C, i.e., no reaction occurs within the curing temperature range of BMI resin (approximately 200 °C). After mixing with BMI resin, the curing reaction of the mixture is delayed to approximately 297 °C, and the peak reaction temperature is delayed to 362 °C. The F2 sizing agent starts to undergo a self-curing reaction at approximately 211 °C, but the reaction enthalpy is only 13.1 J/g, indicating a low degree of chemical reaction, which may be related to its low epoxy value. After mixing with BMI resin, the curing reaction of the mixture is delayed to approximately 265 °C and reaches the peak at approximately 346 °C. The F3 sizing agent starts to undergo self-curing reaction at approximately 207 °C and reaches the peak at approximately 293 °C, with a reaction enthalpy of 183.8 J/g, indicating a high degree of self-curing reaction. After mixing with BMI resin, the mixture starts to react at approximately 213 °C, with a peak temperature of approximately 297 °C and a reaction enthalpy of 225.9 J/g. It is initially judged that the F3 sizing agent can undergo good chemical reactions with the resin within the curing temperature range of BMI resin, which is conducive to the occurrence of strong interfacial chemical bonding.

The reaction characteristics between the three sizing agents and BMI resin were further characterized by in situ FTIR spectroscopy, and the results are shown in Figure 3d–f and S3a–c. In Figure S3a, the infrared characteristic peaks of the F1 sizing agent (especially the epoxy characteristic peak at 916 cm^−1^) show no obvious changes within 50–200 °C, confirming that the F1 sizing agent does not undergo chemical reactions within the reaction temperature range of BMI resin. In Figure 3d, only the characteristic peak of C=C double bonds on the maleimide ring at 947.9 cm^−1^ and the stretching vibration peak of C-H on the aromatic ring in BMI at 3096.6 cm^−1^ gradually disappear with increasing temperature, while the epoxy characteristic peak at 915.1 cm^−1^ shows no obvious changes, indicating that only BMI resin reacts in the mixture and that the F1 sizing agent does not participate in the curing reaction.

In Figure S3b,c, the intensities of the epoxy characteristic peak at 915 cm^−1^ and the -OH vibration peak at 953 cm^−1^ decrease significantly with increasing temperature, indicating that epoxy ring-opening reactions occur in both F2 and F3 sizing agents, which is consistent with the DSC results in Figure 3b,c. In Figure 3e,f, the epoxy characteristic peak at 915 cm^−1^, the -OH vibration peak at 951 cm^−1^, the imide ring stretching peak at 1394.3 cm^−1^, and the C-H stretching peak of the aromatic ring in BMI at 3096.6 cm^−1^ gradually disappear with increasing temperature, suggesting that both the epoxy ring-opening reaction between the curing agent and epoxy groups and the Diels–Alder addition reaction between active hydroxyl groups and bismaleimide rings take place simultaneously in the hybrid system. As a consequence, the contents of epoxy groups and unsaturated imide decrease, and a large number of ether bonds and saturated imide structures are formed in the interfacial region through chemical reactions, resulting in an interfacial transition zone with chemical bonding.

According to the temperature at which the characteristic peaks change, the characteristic peak at 951 cm^−1^ in the F3 sizing agent/BMI mixture shows a significant change at 140 °C, while that in the F2 sizing agent/BMI mixture occurs at 170 °C, indicating that the F3 sizing agent is more likely to react with BMI resin, which is consistent with the DSC results. In summary, the F1 sizing agent does not undergo self-reaction and does not react with BMI resin; the F2 sizing agent undergoes weak self-reaction but reacts with BMI resin; and the F3 sizing agent undergoes self-curing reaction and can react with BMI resin, with higher reaction enthalpy and a more intense reaction. The different reaction characteristics between fiber sizing agents and resin will lead to different interfacial chemical bonding characteristics.

### 3.4. Interfacial Characteristics of T1100-Grade CF/Resin and Their Influence on Interfacial Strength

**Figure 4:**
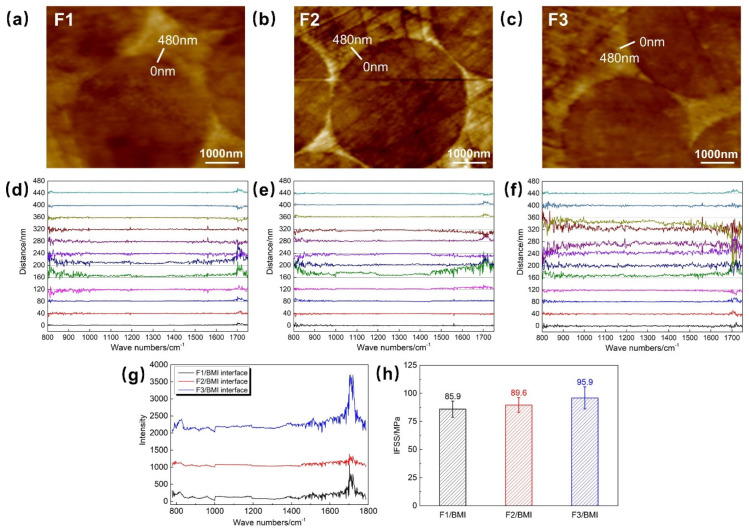
(**a**) Nano-infrared line scanning area diagrams of F1, (**b**) F2, and (**c**) F3 fibers and BMI composites; (**d**) changes in infrared spectra of F1, (**e**) F2, and (**f**) F3 fiber composites with scanning distance in the interfacial region; (**g**) comparison of infrared spectra of the interfacial regions of the three fibers; (**h**) IFSS of the three fibers/BMI resin.

The chemical characteristics of the interfacial region between three T1100-grade CFs and BMI resin were analyzed by nano-infrared spectroscopy. Line scanning of infrared spectra was performed in the interfacial region at an interval of 40 nm, and the line scanning areas are shown in Figure 4a–c. The change in interfacial chemical characteristics with scanning distance was characterized by calculating the difference in infrared spectrum intensity between consecutive scanning points, and the change in spectrum intensity with distance is shown in Figure 4d–f. According to Figure 4d, the spectral intensity of F1 changes slightly within the range of 0–120 nm; at 160 nm, the spectral intensity increases significantly in the positive direction, indicating that the scanning point moves from the fiber region to the interfacial region; at 240 nm, the spectral intensity changes significantly in the negative direction, indicating that the scanning point moves from the interfacial region to the resin region; the spectral intensity changes significantly decreased in the range of 320–440 nm. The thickness of the F1/BMI interfacial region is approximately 80 nm. Using the same judgment method, the interfacial region of F2/BMI is located at 160–320 nm with a thickness of approximately 160 nm (Figure 4f), and the interfacial region of F3/BMI is located at 160–360 nm with a thickness of approximately 200 nm (Figure 4e), which is significantly higher than the approximately 100 nm interfacial zone thickness of conventional CF composites [37]. By comparison, the infrared spectral intensity of the F3 interfacial region fluctuates more significantly, indicating more pronounced changes in the chemical composition of the interfacial region, which may be associated with the more intense chemical reaction between the F3 sizing agent and BMI resin.

The infrared spectra of the interfacial regions of the three composites were compared, and the results are shown in Figure 4g. The positions of the infrared spectral peaks of the three interfacial regions are similar, indicating similar functional group compositions of the interfacial regions. According to calculations, 1 wt% sizing agent can only cover a thickness of approximately 20 nm on the surface of T1100-grade CFs, while the minimum thickness of the interfacial region in the above results is 80 nm, indicating that the resin matrix contributes more significantly to the chemical functional group composition of the interfacial region. In Figure 4g, the infrared spectral intensity of the F3 interfacial region is the highest, followed by F1, and F2 is the lowest. Since the chemical compositions of the three interfacial regions are similar and the infrared spectroscopy test parameters are the same, the spectral intensity can, to a certain extent, represent the concentration of functional groups in the interfacial region. Thus, the F3 interfacial region has the highest functional group concentration, while the F2 interfacial region has the lowest. The concentration of functional groups in the interfacial region is mainly affected by the content of active carbon atoms on the fiber surface. To analyze the influence of the thickness and chemical characteristics of the interfacial region on the interfacial strength of T1100-grade CF/BMI resin, the IFSS of the three CFs with BMI resin was characterized, and the results are shown in Figure 4h. The IFSS of F3 is the highest, reaching 95.9 MPa, followed by F2 (89.6 MPa), and F1 is the lowest (85.9 MPa).

**Figure 5:**
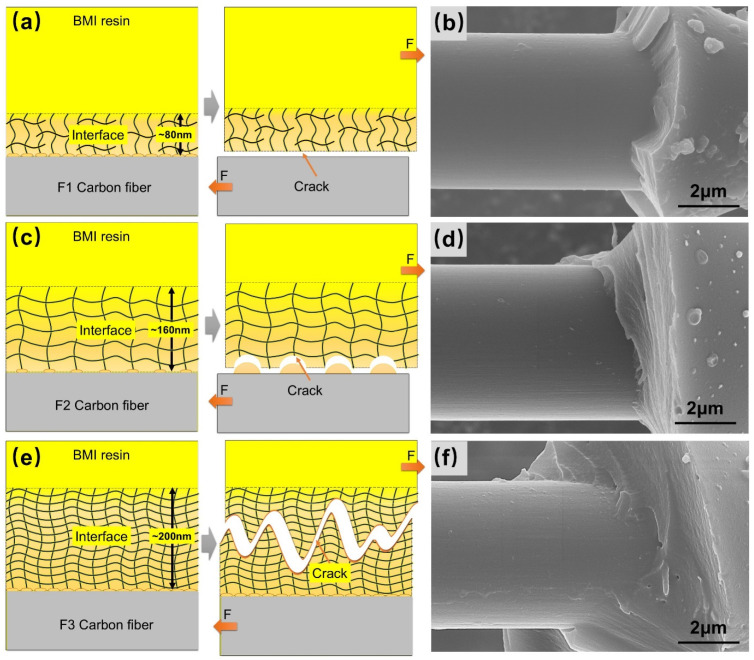
Schematic diagrams of interfacial failure modes of (**a**) F1, (**c**) F2, and (**e**) F3 fibers; SEM images of micro-debonding fracture surfaces of (**b**) F1, (**d**) F2, and (**f**) F3 fibers with BMI resin.

Based on the characteristics of the fracture SEM morphology, the micro-correlation among the fiber surface physical and chemical properties, the reaction characteristics between the sizing agent and BMI resin, and the interfacial failure mode was established. The F1 sizing agent does not undergo chemical reactions with BMI resin within the curing temperature range of BMI resin, resulting in low interfacial chemical bonding strength. Meanwhile, F1 has low surface O atom content and a low O/C ratio, leading to poor wetting of the fiber surface by BMI resin and an interfacial thickness of only approximately 80 nm. F1/BMI resin forms a “thin and weak” interface, and interfacial failure occurs on the CF surface (Figure 5a). The micro-debonding fracture surface of the fiber is smooth with almost no resin residue (Figure 5b), and the IFSS is the lowest.

F2 has a low content of active carbon atoms, resulting in a low concentration of functional groups in the interfacial region. However, the F2 sizing agent undergoes a small amount of chemical reaction with BMI resin, improving the interfacial chemical bonding strength. Compared with F1, F2 has higher surface O atom content and a higher O/C ratio, enhancing the wetting of the fiber surface by BMI resin, and the formed interfacial thickness increases to approximately 160 nm, forming a “relatively thick and strong” interface. Interfacial failure occurs both on the CF surface and the interfacial region adjacent to the fiber surface (Figure 5c). The micro-debonding fracture surface of the fiber is relatively smooth but has a small amount of resin residue (Figure 5d), and the IFSS is improved.

F3 has the highest content of active carbon atoms and thus the highest concentration of functional groups in the interfacial region. Compared with the other two fibers, F3 has the highest surface O atom content and O/C ratio, resulting in the best wetting of the fiber surface by BMI resin. The interfacial thickness formed during resin curing further increases to 200 nm. Meanwhile, the F3 sizing agent undergoes more intense chemical reaction with BMI resin, leading to higher interfacial chemical bonding strength and forming a “thick and strong” interfacial region. Interfacial failure occurs in the interfacial region (Figure 5e), and the micro-debonding fracture surface of the fiber is relatively rough with more resin residue (Figure 5f); the IFSS is the highest.

In summary, the improved interfacial performance of F3/BMI mainly arises from three factors: the higher O/C ratio and oxygen content significantly enhance resin wettability; the F3 sizing agent exhibits stronger reactivity with BMI resin, leading to covalent bonding at the interface; and the thicker interfacial region (~200 nm) relieves stress concentration and inhibits crack propagation. In contrast, the weak interface of F1 is caused by low surface activity, the non-reactive sizing agent with the resin, and a thin interfacial region, which is consistent with the interfacial failure mechanism reported in related studies [35,36,37,38].

### 3.5. Influence of Interfacial Properties on the Performance of T1100-Grade CF/BMI Composites

**Figure 6:**
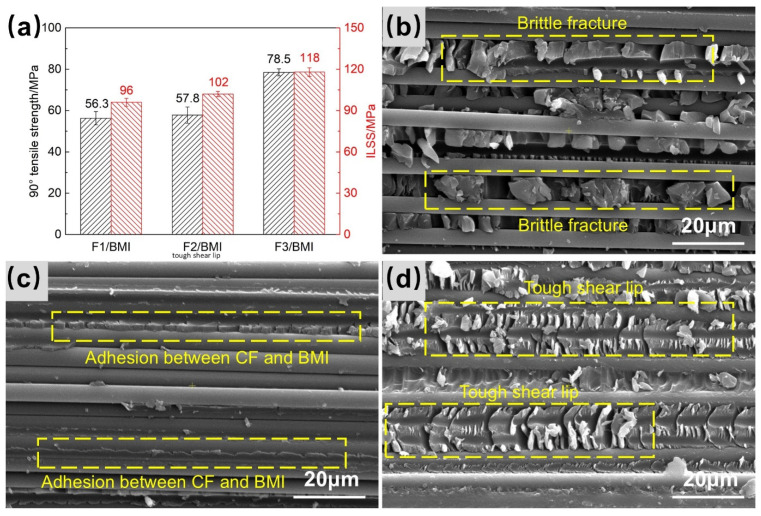
(**a**) The 90° tensile strength and ILSS of three types of composites; SEM images of ILSS fractures of (**b**) F1/BMI, (**c**) F2/BMI, and (**d**) F3/BMI composites.

The influence of interfacial composition and IFSS on the 90° tensile strength and ILSS of T1100-grade CF/BMI composites was further studied, and the results are shown in Figure 6a. The trends of the 90° tensile strength and ILSS of the composites are the same as the IFSS, showing F3 > F2 > F1, presenting a significant correlation. Compared with existing reports [27], the 90° tensile strength of 78.5 MPa and ILSS of 118 MPa obtained in F3/BMI composite are at a high level in the T1100-grade carbon fiber/BMI system. In Figure 6b, the CF surface of the F1 composite ILSS fracture is very smooth with almost no resin residue, and the resin exhibits obvious brittle fracture. There are obvious gaps on the CF surface, which is consistent with the interfacial failure mode shown in Figure 5a, representing typical brittle interface debonding, corresponding to the lowest IFSS (85.9 MPa) and poor mechanical properties. Weak interfacial bonding is prone to cause brittle fracture of the resin, reducing the composite performance. In Figure 6c, the fiber surface of the F2 composite ILSS fracture is also relatively smooth, but there is a certain amount of resin residue on the fiber surface, and some fibers are adhered to the adjacent resin, showing good interfacial bonding. The fracture surface generally presents a failure mode transitioning from brittle to tough, which is a mixed failure mode, corresponding to a moderate IFSS (89.6 MPa). In Figure 6d, the CF surface of the F3 composite fracture is covered with more resin, and the CF is closely combined with the adjacent resin, similar to the schematic diagram in Figure 5e. The resin presents a shear lip morphology of tough fracture, and the high interfacial strength makes the shear failure of the composite show tough fracture, corresponding to the highest IFSS (95.9 MPa) and optimal mechanical properties. Thus, a direct correlation among “microstructure–interfacial bonding–mechanical properties” is established: the stronger the interfacial bonding, the tougher the failure mode and the higher the mechanical properties. Improving the fiber/resin interfacial properties by regulating the fiber surface chemical characteristics helps to transform the composite shear failure from brittle to tough. While exhibiting excellent interfacial properties, the T1100-grade CF/BMI composite in this study was fabricated via the prepreg autoclave molding process, which is a mature, stable, and scalable manufacturing technology in the aerospace field. It is expected to be directly applied to aircraft wings, satellite load-bearing structures, and other scenarios.

## 4. Conclusions

(1) Dry-jet wet-spun T1100-grade CFs exhibit a smooth surface with no obvious grooves and similar surface roughness (approximately 16~17 nm). Thus, the contribution of mechanical interlocking to the CF/BMI interfacial bonding is negligible.
(2) The three T1100-grade CFs display distinct surface chemical properties. F3 shows the highest active carbon content (25.24%), oxygen content, O/C ratio (0.188), and epoxy value, which effectively promote resin wetting and the formation of a thicker interfacial region.
(3) The reactivity between different sizing agents and BMI resin varies considerably. F1 sizing neither cures nor participates in resin curing; F2 sizing shows low self-curing reactivity, with a reaction enthalpy of only 13.1 J/g and weak reaction with the resin; F3 sizing exhibits strong self-curing reactivity, with a reaction enthalpy of 183.8 J/g and high reactivity with the resin, which effectively improves interfacial bonding strength.
(4) The interfacial region was innovatively characterized by nano-infrared spectroscopy. The interfacial thicknesses are approximately 80 nm for F1, 160 nm for F2, and 200 nm for F3. Interfacial shear strength (IFSS) follows the order F3 (95.9 MPa) > F2 (89.6 MPa) > F1 (85.9 MPa), showing a positive quantitative correlation with interfacial thickness and chemical bonding strength.
(5) The quantitative correlation among fiber surface characteristics, interfacial structure, and macro-mechanical properties was established. Constructing a “thick and strong” interfacial region increases IFSS by 11.6% from F1 (85.9 MPa) to F3 (95.9 MPa) and significantly enhances the 90° tensile strength up to 78.5 MPa and interlaminar shear strength (ILSS) up to 118 MPa, driving the failure mode from brittle to ductile.
(6) Sufficient oxygen-containing polar groups and a high O/C ratio are critical to enhancing resin wetting and interfacial compatibility. Abundant epoxy and hydroxyl groups on the fiber surface serve as the key functional groups, which form covalent bonds with BMI resin via epoxy ring-opening and Diels–Alder addition reactions. A high-performance sizing agent should contain sufficient amine curing agent to ensure strong self-curing reactivity and high chemical reactivity with BMI resin, thus contributing to the construction of a “thick and strong” interfacial region and superior interfacial bonding.
(7) The composites show promising applications in aerospace primary load-bearing structures and high-end lightweight equipment. This work promotes the development of high-performance, lightweight, and reliable advanced composites, contributing to lower energy consumption, longer flight endurance, and low-carbon sustainable development of the aerospace industry.

## Figures and Tables

**Table 1:** Reaction conditions of sizing agents of three fibers, BMI resin, and their 1:1 mixtures with BMI resin.

Sample	Curing Onset Temperature (°C)	Peak Temperature (°C)	Enthalpy (J/g)
BMI resin	196.6	260.9	245.1
F1 sizing agent	285.2	351.9	98.1
F1 sizing agent + BMI resin	297.1	362	152.2
F2 sizing agent	210.7	261.2	13.1
F2 sizing agent + BMI resin	264.9	346.3	132.7
F3 sizing agent	206.7	293.8	183.8
F3 sizing agent + BMI resin	213.6	296.8	225.9

## Data Availability

The data supporting the conclusions of this study are not publicly available due to privacy restrictions but are available from the corresponding author upon reasonable request.

## Supplementary Materials

The following supporting information can be downloaded at: https://www.mdpi.com/article/10.3390/polym18070887/s1, Figure S1: Autoclave process schedule diagram; Figure S2: (a) XRD curves of the three fibers; (b) degree of orientation of the three fibers; small-angle X-ray diffraction patterns of (c) F1, (d) F2, and (e) F3; Figure S3: In-situ FTIR spectra of (a) F1 sizing agent, (b) F2 sizing agent, and (c) F3 sizing agent.

## References

[1] Yang H.S., Kim Y.M., Choi H., Jang J., Yu W.R. (2020). Electrochemical wet-spinning process for fabricating strong PAN fibers via an in situ induced plasticizing effect. Polymer.

[2] Das T.K., Ghosh P., Das N.C. (2019). Preparation, development, outcomes, and application versatility of carbon fiber-based polymer composites: A review. Adv. Compos. Hybrid Mater..

[3] Date S., Abe Y., Okabe T. (2022). Effects of fiber properties on aerodynamic performance and structural sizing of composite aircraft wings. Aerosp. Sci. Technol..

[4] Yao S.-S., Jin F.-L., Rhee K.Y., Hui D., Park S.-J. (2018). Recent advances in carbon-fiber-reinforced thermoplastic composites: A review. Compos. Part B Eng..

[5] Dai Z., Shi F., Zhang B., Li M., Zhang Z. (2011). Effect of sizing on carbon fiber surface properties and fibers/epoxy interfacial adhesion. Appl. Surf. Sci..

[6] Karger-Kocsis J., Mahmood H., Pegoretti A. (2019). All-carbon multi-scale and hierarchical fibers and related structural composites: A review. Compos. Sci. Technol..

[7] Hung P.Y., Lau K.T., Fox B., Hameed N., Lee J.H., Hui D. (2017). Surface modification of carbon fibre using graphene–related materials for multifunctional composites. Compos. Part B Eng..

[8] Wang H., Jin K., Tao J. (2020). Improving the interfacial shear strength of carbon fibre and epoxy via mechanical interlocking effect. Compos. Sci. Technol..

[9] Sun Z., Luo Y., Chen C., Dong Z., Jiang G., Chen F., Ma P. (2024). Mechanical enhancement of carbon fiber-reinforced polymers: From interfacial regulating strategies to advanced processing technologies. Prog. Mater. Sci..

[10] Ding R., Sun Y., Lee J., Nam J.D., Suhr J. (2020). Enhancing interfacial properties of carbon fiber reinforced epoxy composites by grafting MXene sheets (Ti_2_C). Compos. Part B Eng..

[11] Zisman W.A. (1969). Surface Chemistry of Plastics Reinforced by Strong Fibers. Ind. Eng. Chem. Prod. Res. Dev..

[12] Sun N., Zhu B., Gao X., Qiao K., Zhang Y., Wang B., Fan J., Yu K., Liu C., Li C. (2023). Improved the interfacial characteristics of carbon fiber/polyamide 6 composites by synthesizing polydopamine rapidly on the carbon fiber surface with ultrasound-assisted. Compos. Sci. Technol..

[13] Li Y., Li H., Dong J., Chen Z., Zhao J., Huan X., Jia X., Ge L., Yang X., Zu L. (2024). Revisiting the sequential evolution of sizing agents in CFRP manufacturing to guide cross-scale synergistic optimization of interphase gradient and infiltration. Compos. Part B Eng..

[14] Zhu J., Song G., Wang C., Li L., Zheng H., Zhang W., Li B., Ma L. (2023). Controllable construction of “degressive gradient” transition layer on carbon fibers surface to enhance interfacial properties of carbon fiber/epoxy composites by layer upon layer assembly of carbon nano tubes. J. Appl. Polym. Sci..

[15] Hu S., Zhu B., Zhang G., Jiang W., Zhou J., Di C., Yu J., Qiao K. (2026). Recent Developments at the Interface of Carbon Fiber-Reinforced Polymer Composites Theory, Modification and Interfacial Properties. Polym. Compos..

[16] Park S.J., Kim B.J. (2005). Roles of acidic functional groups of carbon fiber surfaces in enhancing interfacial adhesion behavior. Mater. Sci. Eng. A.

[17] Wang Z.M., Yamashita N., Wang Z.X., Hoshinoo K., Kanoh H. (2004). Air oxidation effects on microporosity, surface property, and CH4 adsorptivity of pitch-based activated carbon fibers. J. Colloid Interface Sci..

[18] Hui C., Qingyu C., Jing W., Xiaohong X., Liu H., Zhanjun L. (2018). Interfacial enhancement of carbon fiber/nylon 12 composites by grafting nylon 6 to the surface of carbon fiber. Appl. Surf. Sci..

[19] Zhang S., Han P., Yang L., Hu S., Wang J., Gu Z. (2022). Constructing a Double Alternant “Rigid-Flexible” Structure for Simultaneously Strengthening and Toughening the Interface of Carbon Fiber/Epoxy Composites. Nanomaterials.

[20] Sun N., Zhu B., Cai X., Yu L., Yuan X., Zhang Y. (2022). Enhanced interfacial properties of carbon Fiber/Polyamide composites by In-situ synthesis of polyamide 6 on carbon fiber surface. Appl. Surf. Sci..

[21] Kaveh A., Jazani O.M., Jafari M., Ardebili A., Ghaderi H. (2024). Design, preparation and characterization of modified PAN-based carbon fiber by an anodic oxidation process: What is the electrolyte role on the morphology and surface properties?. J. Iran. Chem. Soc..

[22] He D., Yao Y., Cai X.Q. (2011). Effect of anodization on the graphitization of PAN-based carbon fibers of PAN-based carbon fibers. J. Wuhan Univ. Technol.-Mater. Sci. Ed..

[23] Pawar S.S., Hutchinson S.A., Eyckens D.J., Stojcevski F., Hayne D.J., Gengenbach T.R., Razal J.M., Henderson L.C. (2022). Carbon fiber sizing agents based on renewable terpenes. Compos. Sci. Technol..

[24] Bowman S., Jiang Q., Memon H., Qiu Y., Liu W., Wei Y. (2018). Effects of Styrene-Acrylic Sizing on the Mechanical Properties of Carbon Fiber Thermoplastic Towpregs and Their Composites. Molecules.

[25] Liu J., Ge H., Chen J., Wang D., Liu H. (2012). The preparation of emulsion type sizing agent for carbon fiber and the properties of carbon fiber/vinyl ester resin composites. J. Appl. Polym. Sci..

[26] Luo Y., Zhao Y., Duan Y., Du S. (2011). Surface and wettability property analysis of CCF300 carbon fibers with different sizing or without sizing. Mater. Des..

[27] Ren P., Liang G., Zhang Z. (2006). Influence of epoxy sizing of carbon-fiber on the properties of carbon fiber/cyanate ester composites. Polym. Compos..

[28] Hu S., Han P., Meng C., Yu Y., Han S., Wang H., Wei G., Gu Z. (2024). Comparative study of different bonding interactions on the interfacial adhesion and mechanical properties of MXene-decorated carbon fiber/epoxy resin composites. Compos. Sci. Technol..

[29] Zhu C., Li S., Cong X., Rudd C., Liu X. (2021). Effect of Silane Coupling Agent on the Properties of Recycled Carbon Fibers Reinforced Bio-based Epoxy Composites. Fibers Polym..

[30] Yan F., Wang J., Li G., Dai S., Ao Y., Liu L. (2024). Biomimetic mineralization of silicon dioxide onto carbon fiber for elevated interfacial properties and hydrothermal aging resistance of carbon fiber/epoxy composites. Polym. Compos..

[31] Wu Q., Bai H., Zhao R., Ye Z., Deng H., Xiao B., Zhu J. (2022). Amine-caged ZrO2@GO multilayer core-shell hybrids in epoxy matrix for enhancing interfacial adhesion of carbon fiber composites. Compos. Part B Eng..

[32] Quan G., Wu Y., Li W., Li D., Liu X., Wang K., Dai S., Xiao L., Ao Y. (2024). Construction of cellulose nanofiber/carbon nanotube synergistic network on carbon fiber surface to enhance mechanical properties and thermal conductivity of composites. Compos. Sci. Technol..

[33] Hu S., Han P., Zhang S., Wang J., Meng C., Wei G., Gu Z. (2023). Layer-by-layer assembling MXene/poly(3-glycidyloxypropyldimethoxymethylsilane) hierarchical structure onto carbon fibers for high-performance carbon fiber/epoxy composites. Compos. Sci. Technol..

[34] Demchuk Z., Zhu J., Li B., Zhao X., Islam N.M., Bocharova V., Yang G., Zhou H., Jiang Y., Choi W. (2022). Unravelling the Influence of Surface Modification on the Ultimate Performance of Carbon Fiber/Epoxy Composites. ACS Appl. Mater. Interfaces.

[35] Zhang L., De Greef N., Kalinka G., Van Bilzen B., Locquet J.P., Verpoest I., Seo J.W. (2017). Carbon nanotube-grafted carbon fiber polymer composites: Damage characterization on the micro-scale. Compos. Part B Eng..

[36] Egoshi T., Ishikawa T., Tezura M., Kizuka T. (2020). Transmission electron microscopy of unidirectional carbon fiber reinforced plastics at on-axis tension. Materialia.

[37] Yang C., Zhu D., Yang F., Liu Q., Sun C., Lei K., Zheng Z., Wang X. (2020). Quantitative analysis based on atomic force microscopy characterization of interfacial properties between carbon fibers and epoxy resin subjected to hygrothermal and thermal treatments. Compos. Sci. Technol..

[38] Florek P., Król M., Jeleń P., Mozgawa W. (2021). Carbon Fiber Reinforced Polymer Composites Doped with Graphene Oxide in Light of Spectroscopic Studies. Materials.

[39] (2023). Standard Test Method for Tensile Properties of Polymer Matrix Composite Materials.

[40] (2022). Standard Test Method for Short-Beam Shear Strength of Polymer Matrix Composite Materials and Their Laminates.

