# Supplementary material for: Effect of Fiber Surface Characteristics on the Interfacial Properties of T1100-Grade Carbon Fiber Bismaleimide Composites
Source: Polymers (Basel). 2026 Apr 5;18(7):887. doi: 10.3390/polym18070887 (PMC13074581; doi:10.3390/polym18070887)
Supplement: Supplementary file 1 [file polymers-18-00887-s001.zip › polymers-4204583-supplementary.pdf]

Effect of Fiber Surface Characteristics on the Interfacial Properties of T1100-Grade Carbon Fiber Bismaleimide Composites

Tianshu Li, Fenghui Shi *, Weihan Wang, Hongchen Yan, Xiangyu Xu, Baoyan Zhang

AVIC Manufacturing Technology Institute Composite Technology Center, Beijing 101300, China

*Corresponding Author: Fenghui Shi, Email: fenghuishi@126.com

1 Autoclave process

As shown in Fig S1, all composite samples were cured by autoclave process with a curing cycle of 180 °C for 2 h + 200 °C for 6 h and a curing pressure of 0.6 MPa.

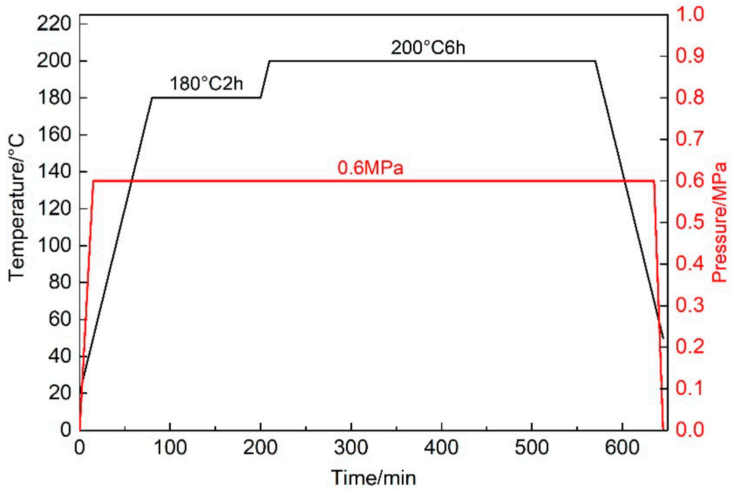

Figure S1. Autoclave process schedule diagram

2 Surface Physical Properties of Different T1100-Grade Carbon Fibers

The X-ray diffraction (XRD) results of the surfaces of three T1100-grade carbon fibers are shown in Supplementary Fig S2(a). The XRD patterns of the three fibers are basically similar. After calculation, the interplanar spacing $d_{[002]}$ of F1, F2, and F3 is 0.3484 nm, 0.3491 nm, and 0.3497 nm, respectively, indicating that the interplanar spacing of the three fibers is comparable. Supplementary Fig S2(b) presents the degree of orientation of F1, F2, and F3 fibers calculated by small-angle X-ray diffraction, which are 0.82, 0.83, and 0.81, respectively, showing similar orientation degrees. Supplementary Fig S2(c), (d), and (e) display the small-angle X-ray diffraction patterns of the three fibers, and their diffraction patterns are basically identical.

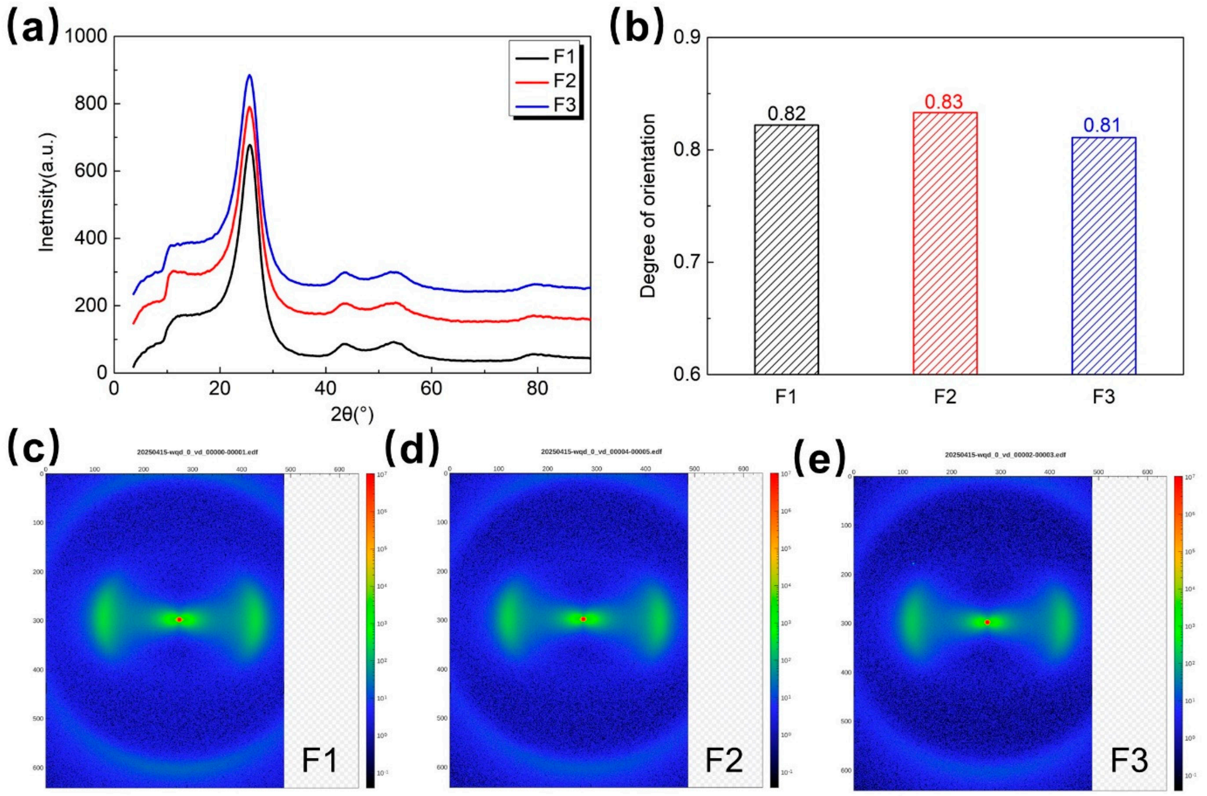

Figure S2. (a) XRD curves of the three fibers; (b) degree of orientation of the three fibers; small-angle X-ray diffraction patterns of (c) F1, (d) F2, and (e) F3.

3 In-Situ Fourier Transform Infrared (FTIR) Spectra of Different Fiber Sizing Agents

The in-situ FTIR spectra of the sizing agents of the three fibers are shown in Supplementary Fig S3. In Supplementary Fig S3(a), the infrared characteristic peaks of the F1 sizing agent (especially the epoxy characteristic peak at 916 cm^{-1}) show no obvious changes within the range of 50–200 °C, confirming that the F1 sizing agent does not undergo chemical reactions within the reaction temperature range of the bismaleimide resin. In Supplementary Fig S3(b) and (c), the intensity of the epoxy characteristic peak at 915 cm^{-1} and the -OH vibration peak at 953 cm^{-1} decreases significantly with increasing temperature, indicating that the sizing agent undergoes an epoxy ring-opening reaction. The -OH vibration peak at 953 cm^{-1} in Supplementary Fig S3(c) basically disappears at 140 °C, while that in Supplementary Fig S3(b) basically disappears at 170 °C. This indicates that the F3 sizing agent has a lower initial reaction temperature than the F2 sizing agent and is more prone to self-curing reaction.

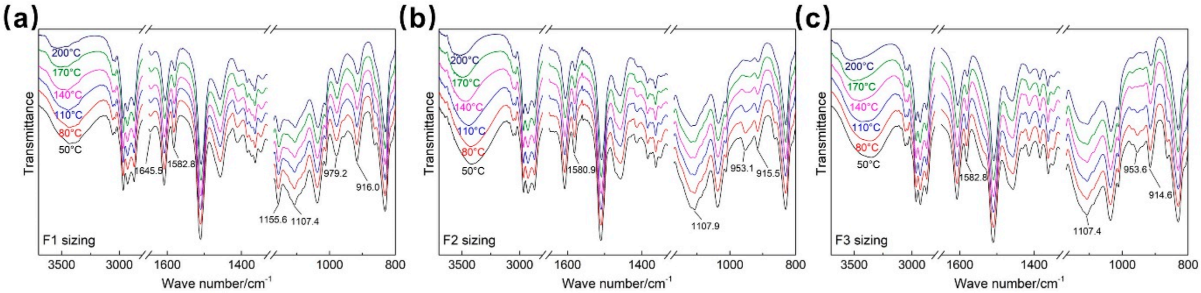

Figure S3. In-situ FTIR spectra of (a) F1 sizing agent, (b) F2 sizing agent, and (c) F3 sizing agent.
